# 

**Published:** 1904-05

**Authors:** 


May, 1904. VOL. XXV. No. 5.

### THE INTERNATIONAL DENTAL JOURNAL.

LIBRARY
INDEXED
G. O.

JAMES TRUMAN, D.D.S., EDITOR,
PHILADELPHIA.

*COLLABORATORS:*

WILLIAM H. POTTER, A.B., D.M.D., BOSTON.
CHARLES P. BRIGGS, A.B., M.D., D.M.D., BOSTON.

LIBRARY
SURGEON GENERAL'S OFFICE
MAY -3 1904

### Summary of Contents.

(*For details, see p. 2.*)

ORIGINAL COMMUNICATIONS.

REPORTS OF SOCIETY MEETINGS.

DOMESTIC CORRESPONDENCE.

EDITORIAL.

MISCELLANY

CURRENT NEWS.

REVIEWS OF DENTAL LITERATURE.

*$2.50 a Year, in Advance. Single Copies, 25 Cents.*

PUBLISHED MONTHLY BY

The International Dental Publication Company,

227-231 SOUTH SIXTH ST., PHILADELPHIA.

EDITORIAL OFFICE, 4505 CHESTER AVENUE, PHILADELPHIA, PA.

PRINTED BY J. B. LIPPINCOTT COMPANY, PHILADELPHIA.

Entered at the Post-Office at Philadelphia, and admitted for transmission through the mails at second-class rates.

